# Expected and Unexpected Features of the Newly Discovered Bat Influenza A-like Viruses

**DOI:** 10.1371/journal.ppat.1004819

**Published:** 2015-06-04

**Authors:** Wenjun Ma, Adolfo García-Sastre, Martin Schwemmle

**Affiliations:** 1 Department of Diagnostic Medicine/Pathobiology, Kansas State University, Manhattan, Kansas, United States of America; 2 Department of Microbiology, Icahn School of Medicine at Mount Sinai, New York, New York, United States of America; 3 Global Health and Emerging Pathogens Institute, Icahn School of Medicine at Mount Sinai, New York, New York, United States of America; 4 Department of Medicine, Division of Infectious Diseases, Icahn School of Medicine at Mount Sinai, New York, New York, United States of America; 5 Institute of Virology, University Medical Center Freiburg, Freiburg, Germany; University of Kentucky, Lexington, UNITED STATES

Influenza A viruses (IAVs) are important zoonotic pathogens that cause epidemic outbreaks in poultry, wild birds, swine, and other mammals. In humans, IAVs cause severe respiratory illness, and zoonotic transmission of IAVs from avian reservoirs poses a constant threat to the public health, as exemplified by the recent outbreak of an avian IAV of the H7N9 subtype [1]. Aquatic birds are considered to be the major reservoir of IAVs, and 16 hemagglutinin (HA) and nine neuraminidase (NA) viral subtypes have been isolated from avian species to date. It is now well documented that from time to time IAVs overcome the species barrier and establish new lineages in other animals, including domestic animals, sea mammals, and humans (Fig 1). Our understanding of IAVs was recently challenged by the identification of two novel genome sequences of influenza A-like viruses from bat specimens by next-generation sequencing. These viruses were provisionally designated “H17N10” (from yellow-shouldered fruit bats [*Sturnira lilium*] in Guatemala) and “H18N11” (from flat-faced fruit bats [*Artibeus planirostris*] in Peru) [2,3], which might signal an expansion of IAV host range (Fig 1).

**Fig 1:**
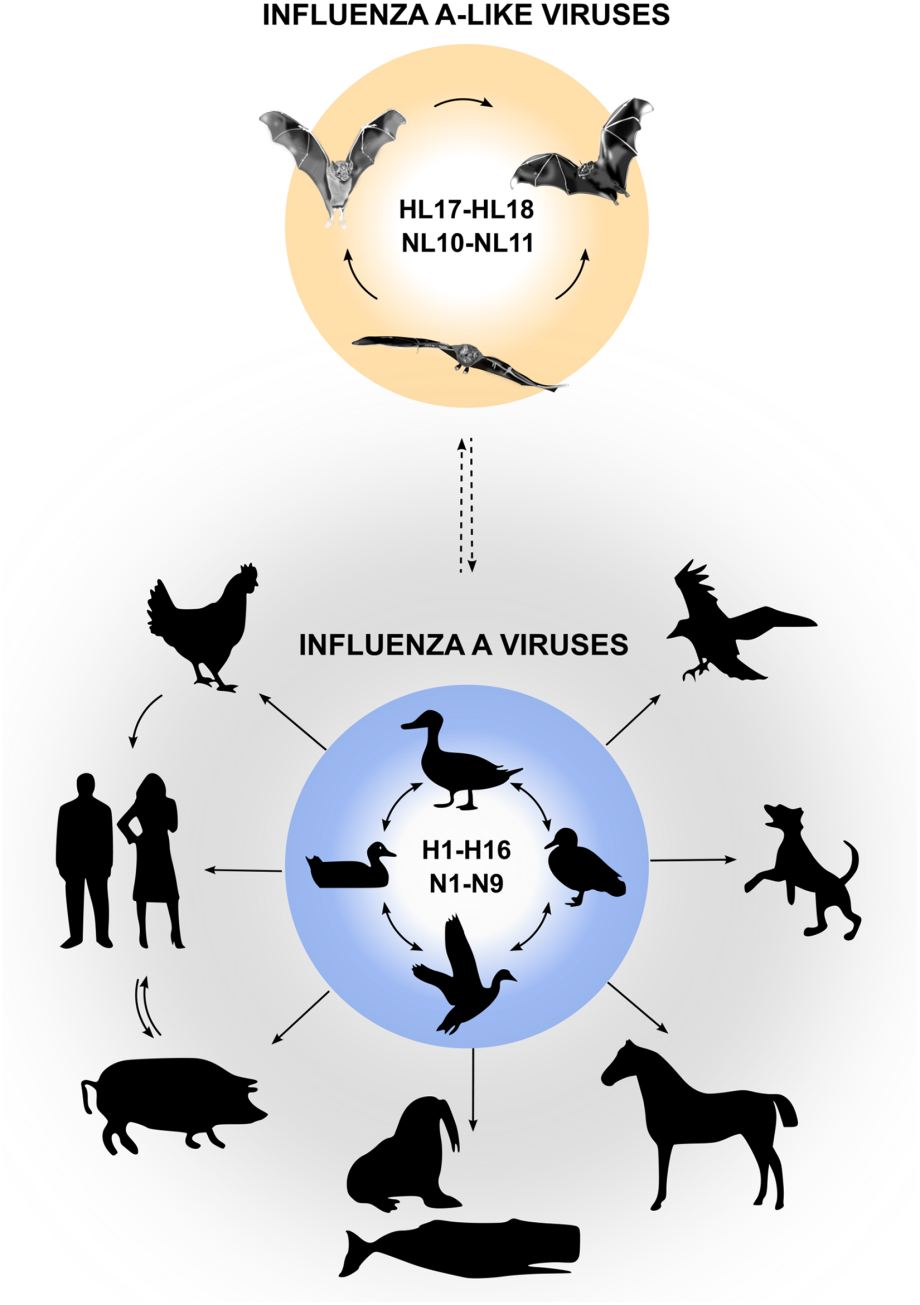
Reservoirs of IAVs and bat influenza-A-like viruses. Natural reservoirs of classical IAVs are wild water birds, from which they can be transmitted to a wide variety of other species. Bat influenza A-like viruses are likely to circulate in various bat species in Central and South America and possibly originate from classical IAVs (indicated by dashed arrow).

## Are Bat Influenza A-like Viruses Different from Classical IAVs?

Biochemical and structural studies indicate that both the influenza A-like H17 and H18 proteins do not bind canonical sialic acid—containing receptors [3–5], which are bound by HAs of conventional IAVs for initiation of infection. Furthermore, the crystal structure of H17 and H18 revealed that these proteins possess a distorted putative sialic acid binding site and showed low thermostability when compared to all known well-characterized HAs [3,5]. In fact, H17 and H18 HAs are unable to bind and hemagglutinate red blood cells and therefore are not “true” HAs. Thus, we suggest that HAs from both H17 and H18 influenza A-like viruses should be named as “HA-like” (HL) proteins (HL17 and HL18).

Consistent with the observation that bat influenza A-like HL17 and HL18 do not bind to canonical sialic acid receptors, bat NAs lack detectable neuraminidase activity [6]. Although the overall N10 structure is similar to other known influenza NA structures, the region corresponding to the highly conserved active site in the N1–N9 subtypes is substantially different [6,7]. The structural features and the fact that the recombinant N10 protein exhibits no or extremely low NA activity suggests that it may have a different function than the NA proteins of other influenza viruses. We therefore suggest that N10 and N11 from bat influenza A-like viruses should be designated as “NA-like” (NL) proteins (NL10 and NL11). Furthermore, the current classification of bat influenza A-like viruses “H17N10 and H18N11” is misleading and should be reconsidered; we suggest that they can be designated as “HL17NL10 and HL18NL11”. At this moment, it is even unclear whether the receptor binding protein is the HL or the NL protein.

## Bat Internal Proteins Are Similar to Those of Extant IAVs

Unlike the bat influenza A-like virus surface proteins, some of the internal proteins of HL17NL10 and HL18NL11 seem to be highly compatible with conventional IAVs [8,9]. This is mainly based on results from viral polymerase reconstitution experiments of a broad variety of IAVs, including the H1N1, H3N2, H5N1, and H7N9 subtypes [8–10]. In all cases, unimpaired polymerase activity was observed after substitution of the conventional IAV nucleoprotein (NP) with bat influenza A-like NP from either HL17NL10 or HL18NL11. Similarly, the polymerase subunit PB2 of both bat influenza A-like viruses partially supported the polymerase activity of some IAVs [8–10]. Internal proteins of HL17NL10 and HL18NL11 are fully compatible between each other, including the polymerase subunits [8]. Recently, the crystal structure of the bat influenza A-like virus polymerase complex was solved, providing a more detailed understanding of viral replication and transcription processes of bat influenza A-like viruses and IAVs in general [11]. This might be especially interesting for investigation of the polymerase compatibility between classical IAVs and bat influenza A-like viruses, as some of the polymerase subunits are interchangeable, whereas others are not.

Functional compatibility of the internal proteins of influenza A-like HL17NL10 with IAV was also observed in the formation of infectious virus-like particles (VLPs) of a conventional IAV [9]. Infectious VLPs of conventional IAVs could be reconstituted with NP, matrix protein 1 (M1), or combinations of HL17NL10 proteins such as the polymerase complex, or M1 and M2, or all internal proteins. Therefore, the internal proteins of bat influenza A-like viruses seem to be functionally equivalent to conventional IAV proteins. This functional compatibility seems to be restricted to IAVs, since bat influenza A-like NPs, as well as the polymerase subunits, do not support the polymerase activity of influenza B viruses [8].

## Are Bats a Reservoir for Bat Influenza A-like Viruses and Classical Influenza A Viruses?

Serological surveys indicate that influenza A-like viruses of the HL17 or HL18 subtypes circulate in various bat species in Central and South America, including predominantly *Sturnira* sp., *A*. *planirostris*, *Artibeus lituratus*, *Carollia perspicillata*, *Myotis* sp., *Molossus molossus*, and others (Fig 1) [3]. These studies also demonstrated that up to 50% of tested samples collected from different bat species in South America are seropositive to HL18 or NL11, and specific antibodies to HL17 are found in 38% of samples collected from nine bat species in Central America [3], suggesting that widespread circulation of bat influenza A-like viruses exists among bats in Central and South America. However, formal proof of transmission events between bat species is missing. Whether bats are infected with other influenza A-like virus subtypes is currently unknown. Moreover, it also remains unclear whether bat influenza A-like HL17NL10 and HL18NL11 viruses can be found outside of Central and South America. Based on a reverse transcription PCR (RT-PCR) screen that allows detection of HL17NL10 only, central European bats seem to be free of this influenza A-like virus subtype [12]. Although classical IAVs can be isolated from bats in rare cases and antibodies against IAVs were also detected in bats [13], there is no evidence for the infection of New World bats with classical IAVs of the H1 and H5 subtype [3].

## How Can We Study These Bat Influenza A-like Viruses?

**Fig 2:**
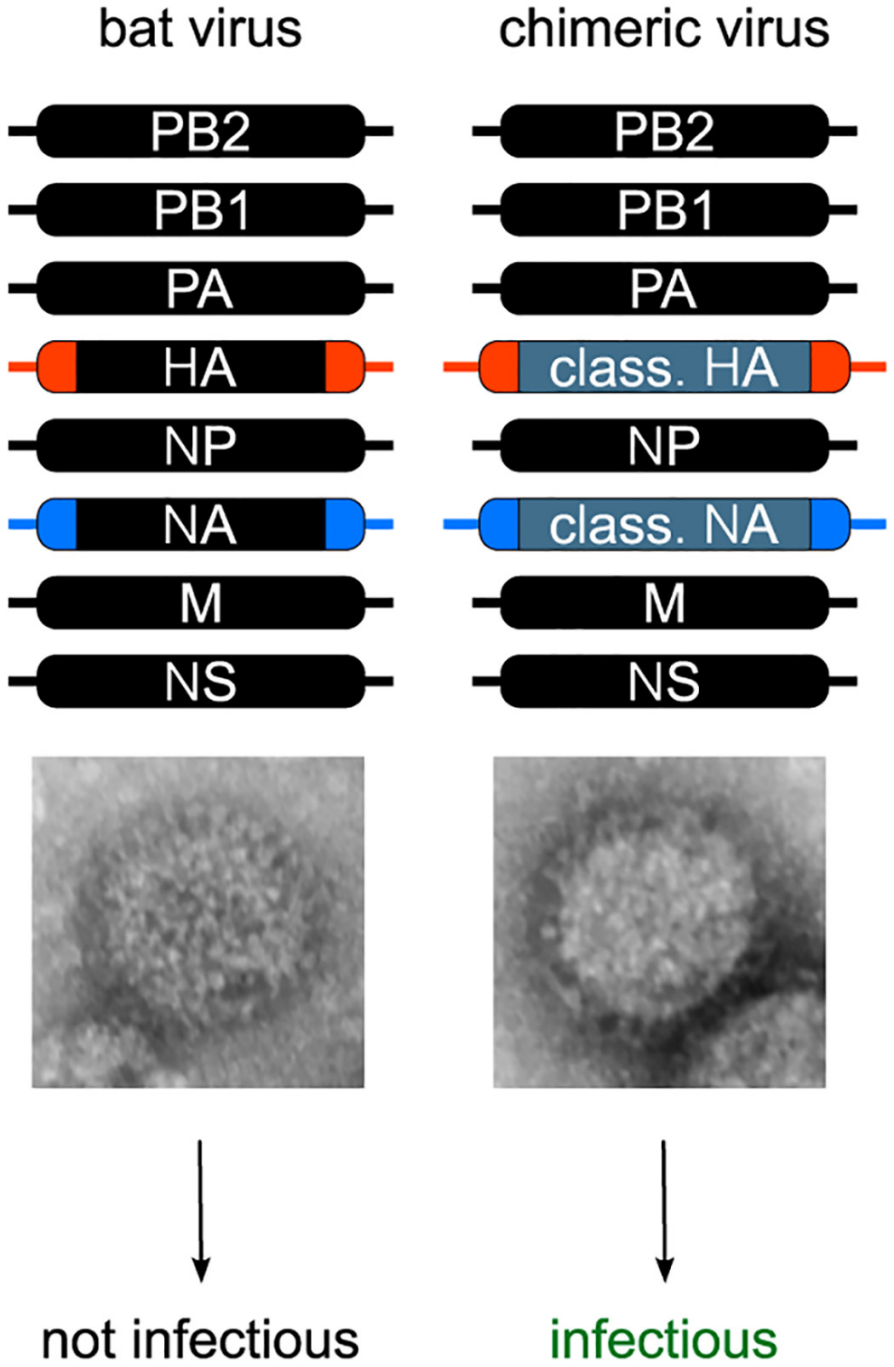
Generation of recombinant bat chimeric influenza viruses. Although rescue attempts with a complete authentic set of either HL17NL10 or HL18NL11 genome segments resulted in the release of viral particles as evidenced by electron microscopy (HL17NL10 particle is shown), no viral growth was observed in various cell culture systems. In contrast, recombinant bat chimeric viruses encoding HA and NA of classical IAVs were highly infectious. Successful rescue of bat chimeric viruses requires bat virus specific packaging sequences (highlighted in blue and red), including the noncoding region and part of the bat virus gene segment 3′ and 5′ open reading frame.

All efforts to isolate an infectious virus from bat species have failed to date, although HL17NL10 or HL18NL11 RT-PCR-positive tissue and rectal swab samples were used in these studies [2,3]. The challenge in isolating infectious influenza A-like viruses could also be related to the atypical surface proteins of these viruses, especially since there is no evidence that the cell lines used for virus isolation, including bat, human, canine, and avian cells [2,3,8,9], express the receptors that allow viral particle uptake. It might be then not surprising that the generation of wild-type influenza A-like HL17NL10 or HL18NL11 viruses using reverse genetic approaches has not succeeded. However, from these rescue attempts, we learned by electron microscopy studies that viral particles are released from human HEK293T cells (Fig 2) [8]. Thus, subsequent binding and internalization of the viral particles in different cells that are normally used for the amplification of recombinant IAV might be impaired.

Recently, we succeeded in generating chimeric bat viruses in mammalian cells containing six internal genes from either influenza A-like HL17NL10 or HL18NL11 virus, with the remaining two surface genes encoding the HA and NA from a conventional IAV such as A/PR/8/1934 (H1N1), A/swine/Texas/4199-2/1998 (H3N2), or A/SC35M (H7N7) [8,9]. To rescue these bat chimeras, it was essential to flank the canonical IAV HA and NA coding regions with the noncoding regions and part of the coding regions from the bat influenza A-like HA (HL17 or HL18) and NA (NL10 or NL11) segments, indicating RNA packaging incompatibilities between bat influenza A-like virus and conventional IAV segments (Fig 2). The major packaging sequences of conventional IAV are located within these regions [14,15] and vary only slightly, thereby allowing reassortment of viral genomes between different IAVs [16]. For efficient growth of bat chimeric viruses in mammalian cells or mice, adaptive mutations are not necessarily required. However, viral replication and pathogenicity in mice is dependent to a certain degree on the HA/NA combination used [8]. The HL17NL10-based bat chimeric virus with the viral surface glycoproteins of a mouse adapted H7N7 virus (A/SC35M) shows limited replication and no pathogenicity in mice [9], while mice infected with a comparable virus dose of the chimera with HA and NA coding regions from A/PR8/H1N1 died from extensive lung pathology, typically observed with conventional IAV [8]. Whether this is due to an intrinsic feature of the different HA/NAs or the interplay between viral surface glycoproteins and internal genes of the bat influenza A-like viruses remains to be determined.

## What Is the Zoonotic Potential of These Bat Influenza A-like Viruses?

Several lines of evidence indicate that bat influenza A-like viruses are of low risk for the human population. This is based on the observation that viral particles of HL17NL10 or HL18NL11 generated by reverse genetics failed to productively infect human cells, which correlates with the general inability of the bat HAs and NAs to mediate cell entry into these cells [3,4,7]. An important feature of an IAV is the possibility to exchange genomic RNA segments with other IAVs, resulting in novel genotypes and phenotypes with zoonotic potential. However, coinfection experiments demonstrated no reassortment between bat chimeric viruses and conventional IAVs [8,9]. The larger sequence diversity between bat influenza A-like virus and conventional IAV genomes most likely caused this packaging incompatibility between these viruses. In addition, specific incompatibilities at the protein level might further prevent the emergence of reassortant viruses after coinfection occurrence. This especially includes the bat influenza A-like polymerase subunits PB1 and PA, since these two proteins do not support the polymerase activity of conventional IAVs [8,9].

## Future Directions

Despite considerable progress, there are many questions that remain to be addressed: How can we propagate infectious viruses in vitro for further studies? What is the receptor and is it specific for bats? Are there more influenza and influenza-like viruses circulating in bats or other hosts in Central and South America and other parts of the world? If so, do these viruses pose a risk for domestic animals and/or humans as observed in bat-derived severe acute respiratory syndrome (SARS), rabies, Nipah, and probably Ebola viruses?
